# Heterotopic Quadruplet Pregnancy. Literature Review and Case Report

**DOI:** 10.3390/medicina57050483

**Published:** 2021-05-12

**Authors:** Brîndușa Cimpoca, Amira Moldoveanu, Nicolae Gică, Corina Gică, Anca Marina Ciobanu, Anca Maria Panaitescu, Dana Oprescu

**Affiliations:** 1 Department of Obstetrics and Gynecology, Carol Davila University of Medicine and Pharmacy, 020021 Bucharest, Romania; brindusa.cimpoca@yahoo.com (B.C.); anca.panaitescu@umfcd.ro (A.M.P.); dana.oprescu@hotmail.com (D.O.); 2 INSMC “Alessandrescu Rusescu”, 011062 Bucharest, Romania; amira.moldoveanu@yahoo.com; 3 Department of Obstetrics and Gynecology, Filantropia Clinical Hospital, 011171 Bucharest, Romania; mat.corina@gmail.com (C.G.); ciobanu.ancamarina@gmail.com (A.M.C.)

**Keywords:** heterotopic quadruplet pregnancy, quadruplet intrauterine and ectopic pregnancy, synchronous intrauterine and ectopic pregnancy

## Abstract

Heterotopic pregnancy is the condition where both intrauterine and ectopic pregnancy are present. It rarely occurs after natural conception, but is more common with assisted reproductive techniques, when more than one embryo is transferred. Quadruplet heterotopic pregnancy is exceedingly rare. Methods: A literature review was conducted aiming to highlight the diagnosis difficulties and the management options in heterotopic quadruplet pregnancies. Results: Nine relevant studies were identified by researching PubMed up to 2021 for “heterotopic quadruplet pregnancy”, “quadruplet intrauterine and ectopic pregnancy”, “synchronous intrauterine and ectopic pregnancy”. Conclusions: In this paper, we present a case of heterotopic quadruplet pregnancy and address the difficulty in diagnosing this condition and make formal recommendations.

## 1. Introduction

Heterotopic pregnancy is the condition where both intrauterine and ectopic pregnancies are present. It rarely occurs after natural conception, but is more common in IVF (in vitro fertilization), when more than one embryo is transferred [1]. Although ectopic pregnancy is not uncommon in women of reproductive age, heterotopic pregnancy is a rare event, and the incidence varies from 1/8000 to 1/30,000 [2,3]. In general practice, ectopic pregnancy is rarely considered if intrauterine pregnancy is confirmed. The increased incidence of pelvic inflammatory disease, the wide use of ovarian stimulation with or without assisted reproductive techniques (ART) have contributed to the increasing incidence of both, multiple gestations and heterotopic pregnancies over the last decades [4]. Quadruplet heterotopic pregnancy may occur when there is simultaneously a pair of intrauterine twins and another pair of twins outside the uterine cavity. At times there is an intrauterine triplet pregnancy and a singleton ectopic gestation.

The aim of this paper is to offer a narrative literature research regarding diagnosis and management options for heterotopic quadruplet pregnancy and to present a case managed in our units.

## 2. Materials and Methods

A systematic research of the literature was conducted in the database of PubMed, in order to select full-length articles published in peer-reviewed journals up to February 2021. The keywords included in the search strategy were: Heterotopic quadruplet pregnancy, quadruplet intrauterine and ectopic pregnancy, synchronous intrauterine, and ectopic pregnancy.

## 3. Results

We found a total number of 15 relevant articles, published between 1989 and 2021. We selected only full-text articles, with studies including a population of women aged 18 years and older, published in this period in the literature and only nine fulfilled the criteria of truly quadruplet heterotopic pregnancy (Table 1).

Almost all pregnancies included in the case reports were conceived with assisted reproductive techniques and only three out of nine had an unremarkable previous obstetric history. Heterotopic quadruplet pregnancy usually appears after ART and ovulation induction. The risk is 30–60 times higher after assisted reproduction when compared to spontaneous conception [14]. All cases included twin or triplet intrauterine pregnancy. The diagnosis was most of the time clinically guided, as severe pain was the predominant symptom. In eight out of nine cases, the diagnosis was made in the first trimester. Interestingly, Aguemon CT et al. reported the case of a late diagnosis, the reason being that this was the case of an abdominal pregnancy that developed until almost term [12].

There are several treatment options for ectopic pregnancy [15]. When there is a heterotopic pregnancy diagnosed, local treatments are preferred. Local methotrexate injection or local potassium chloride injection (KCl) are the topical medical treatments available. However, when heterotopic pregnancy is considered, methotrexate is not the elective choice due to the toxicity for the intrauterine pregnancy [16]. Therefore, KCl injected in the ectopic sac is recommended. In the searched literature, only one case managed the heterotopic pregnancy with local injections. Seven out of the nine papers reviewed managed the cases surgically. The non-medical treatment consists of laparoscopy or laparotomy aiming for removal of the ectopic pregnancy, leaving the intrauterine gestation untouched [6].

## 4. Case Presentation

We present the case of a 35-year-old woman, with history of primary infertility but otherwise healthy. The patient was under the care of reproductive medicine services for male factor infertility with severe oligo-asteno-teratospermia. The pregnancy was obtained after an IVF treatment, with an antagonist short protocol (recombinant FSH 200 UI/day for 10 days and gonadotrophin releasing hormone antagonist for 5 days). On the day of oocyte retrieval she had 20 follicles above 14 mm, but only 6 oocytes were obtained. Intracytoplasmic sperm injection (ICSI) was performed using the only 5 mature oocytes (MII). On the next day, there were only four normally fertilized embryos (2PN). She had day 3 embryo-transfer (ET) of four embryos (one 8-cell embryo grade 1, one 8-cell embryo grade 2, and two 4-cell embryos grade 2–3). She had luteal phase support with vaginal micronized progesterone 400 mg twice daily. Her first βhcg level was 601 mIU/mL and progesterone 46.7 ng/mL. After 48 h, the βhcg level was 1383.28 mIU/mL. She had the first ultrasound examination 14 days after the βhcg dynamic, with the visualization of a triplet pregnancy. The follow-up was programed in 10 days.

**Figure 1:**
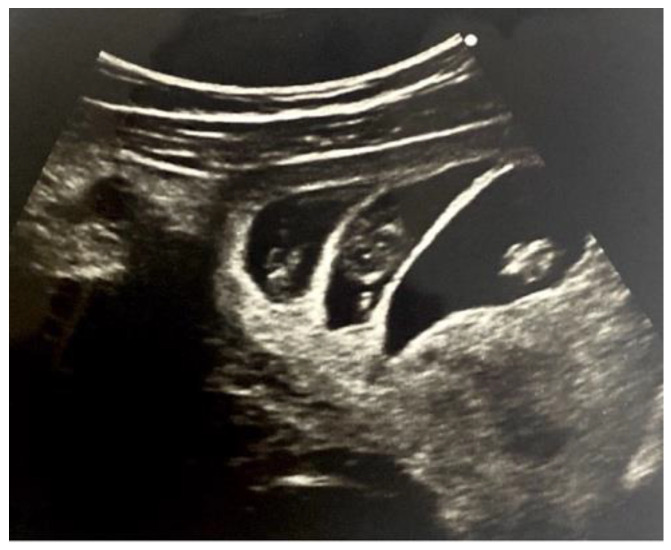
Transabdominal scan at 9 weeks of pregnancy confirms a trichorionic triamniotic (TCTA) pregnancy. Clear visualization of the two lambda signs and thick intertwin membranes, composed of a central layer of chorionic tissue sandwiched between two layers of amnion, therefore multi-layered, more echogenic.

In early pregnancy, at the 9 weeks of gestation, after ET the ultrasound scan demonstrated a triplet triamniotic viable intrauterine pregnancy was diagnosed (Figure 1) along with ovarian hyperstimulation syndrome. She was hemodynamically stable, however at 10 weeks gestational age she complained of lower abdominal pain.

**Figure 2:**
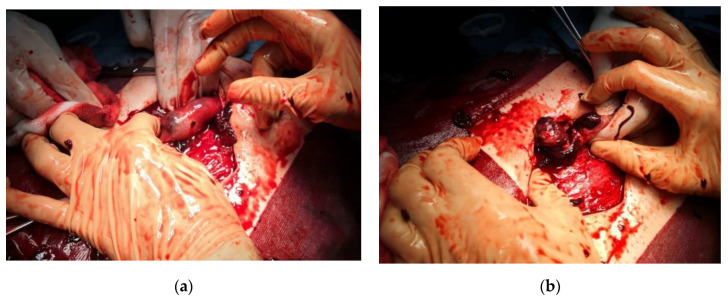
Intraoperative aspects: (**a**) Direct visualization of the ruptured fallopian tube during laparotomy; (**b**) unilateral salpingectomy was performed, and overall blood loss was 1500 mL.

On physical examination, the patient’s vitals were stable, and there was tenderness in the left lower abdomen. Transabdominal ultrasound revealed free intraperitoneal fluid, raising the question of ruptured ectopic pregnancy. Hemoglobin abruptly decreased from 9.5 to 7.5 g/dL and she experienced dyspnea, therefore exploratory laparotomy was performed (Figure 2a,b) and revealed intraperitoneal blood accumulation (approximately 1500 mL), dilated ruptured right fallopian tube measuring 10/4 cm and hyperstimulation aspect of the right ovary. Unilateral salpingectomy was performed and the patient had an uneventful recovery and was discharged on day 3.

The post-surgical evolution was uneventful, the mother continued progesterone treatment as per the guidelines. At the first trimester screening and early anomaly scan (12 weeks) only two of the TCTA fetuses were alive and the pregnancy evolved as a DCDA twin pregnancy, with a low risk for aneuploidies. Serial measurements for cervical length were arranged and the patient was followed up according to our local protocol for DCDA twins. The rest of the pregnancy was completely uneventful. She delivered via cesarean section due to the transverse presentation of the fetus A at 35 weeks after a spontaneous rupture of the membranes, two girls (2200 and 2100 g, Apgar 9 and 10 at 1 and 5 min), without structural defects.

## 5. Discussion

The diagnosis of heterotopic pregnancy is sometimes difficult to make, but one might consider it when there is acute abdominal pain and hemorrhagic shock, in the presence of an intrauterine pregnancy, especially after ART. New onset acute abdominal pain and hemorrhagic shock sings are formal indications for laparoscopy or laparotomy in any pregnant patient.

**Figure 3:**
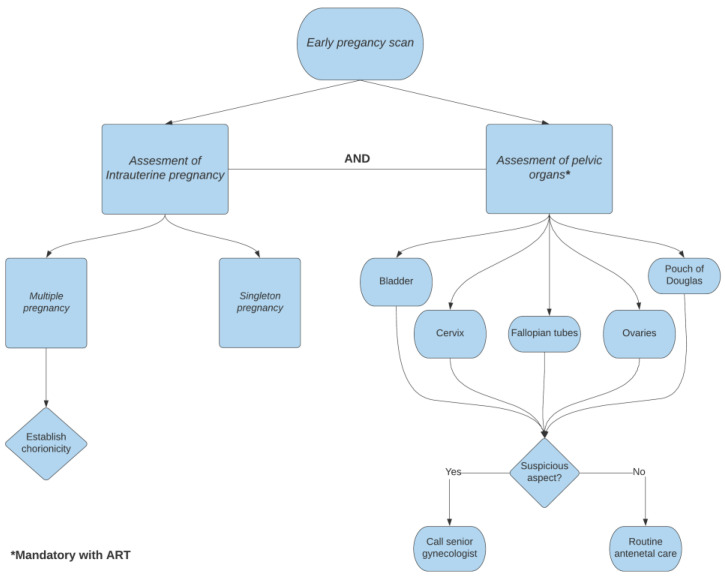
Flowchart on decision making after confirming an intrauterine pregnancy and assessment of pelvic organs.

Aiming to avoid the late diagnosis of a heterotopic pregnancy, and therefore rupture of the tube and surgical repair, we propose a new approach. We recommend systematic transvaginal ultrasound assessment of the female pelvis after diagnosing an early intrauterine pregnancy for high risk patients (IVF procedures and ovarian stimulation). This should be arranged particularly for the high-risk population for developing heterotopic pregnancy with a systematic approach that must be followed at the early pregnancy (6–7 weeks of gestation) scan. After an intrauterine pregnancy is confirmed, the sonographer must visualize the bladder and the relationship between the cervix and the uterus must be established. One must routinely visualize the ovaries and fallopian tubes. Should there be latero-uterine images, suspicious aspect of the fallopian tubes or considerable amount of free fluid in Douglas pouch, the possibility of heterotopic pregnancy should be raised. Eventually, a senior gynecologist (if available) can confirm the diagnosis and local transvaginal KCl injection in the ectopic pregnancy could be administered, aiming to avoid surgical treatment. Transvaginal ultrasonographically-guided injection of KCl (1 mL of 2 mEg/mL), using a 17-gauge needle is performed under general anesthesia, directly into the fetal thorax. The procedure aims to arrest the cardiac activity in the fetus [17]. An overview of the algorithm is pictured in Figure 3. Regarding the number of embryos transferred, the ESHRE guideline recommends a single embryo transfer and this decision should be made by the quality of the embryo, the stage of development, maternal age, ovarian response, and rank of treatment, but to transfer more than two embryos is discouraged [18]. An early ultrasonographic diagnosis of multiple pregnancies is mandatory, in order to establish a normal intrauterine localization, chronicity, and to detect different abnormal major structural defects. In extremely rare cases, the late splitting of the embryonic mass in two can lead to a conjoined twin pregnancy, with its major complication [19].

## 6. Conclusions

We acknowledge that the heterotopic pregnancy is a rare event, and therefore the learning curve for an accurate diagnosis can take a long time. We propose a systematic approach of the pelvis in high risk patients, with every early pregnancy ultrasound in order to identify the extremely rare cases of heterotopic pregnancy.

## Figures and Tables

**Table 1:** Characteristics of included-case reports.

Author	Age (y)	Past History	GA(w)	Method	Intrauterine	Ectopic	Symptoms	Intervention	Obstetric Outcome
Park HR [5]	30	None	NA	NA	Twins	Cornual and tubal	NA	-cornual pregnancy: US guided transvaginal injection of KCl -tubal pregnancy laparoscopy	Elective CS at 37 w twins
Chan [6]	31	bilateral salpingectomy	7	IVF	Twins	interstitial twins	painless vaginal spotting 35 days after ET	Laparotomy	Elective CS at 38 w twins
Tamhane NA [7]	32	Medically managed ectopic pregnancy	10	ICSI	TCTA triplets	Tubal	abdominal pain, vaginal spotting	-10 weeks: Tubal pregnancy- laparotomy -12 weeks: ER triplets to twins	CS after PPROM at 34 w twins
Soares A [8]	31	Primary infertility	9	Ovulation induction	Triplets only 1 viable	Tubal	abdominal pain	Laparotomy	Term delivery singleton-agenesis of one distal phalanx of the hand and agenesis of distal phalanges of all toes
Omosh RK [9]	20	Primary infertility	10	Ovulation induction + IUI	Triplets	Tubal	abdominal pain	Laparotomy	NA pregnancy outcome; at 23 weeks all 3 viable
Uysal F [10]	30	Primary infertility	7	IUI	Triplets	Tubal	abdominal pain	-7 weeks: Laparoscopy -13 weeks: 1 spontaneous fetal loss	Elective CS at 37 w twins
Sherer DM [11]	32	Unremarkable	8	IVF	Triplets	Interstitial	abdominal pain, anemia, marked weakness, and right shoulder pain	Laparoscopy	CS after PPROM at 33 w triplets
Aguemon CT [12]	22	Unremarkable	34	Spontaneous	TCTA triplets	Abdominal	severe preeclampsia + periumbilical pain	Laparotomy and CS -abdominal: Multiple malformation alive; + administration of methotrexate -livebirth of TCTA triplets	3 Neonatal deaths, 1 survivor Maternal heart failure- 6 months F/U stable
Lavanya R [13]	NA	Primary infertility	12	Ovulation induction	Twins	Twins	abdominal pain, vaginal bleed	None viable intrauterine twin + methotrexate	None
Our case	35	Primary infertility	10	Ovulation induction + IVF	TCTA triplets	tubal	abdominal pain	Laparotomy 12 weeks: Only 2 fetus alive	CS after PPROM at 35 w twins

NA—not available; US—ultrasound; CS—cesarean section; IVF—In vitro fertilization; ET—embryo transfer; ICSI intracytoplasmic sperm injection; TCTA—Trichorionic Triamniotic Triplets; ER—embryo reduction; PPROM—preterm premature rupture of membrane; IUI—intrauterine insemination; F/U—follow-up.

## Data Availability

The data presented in this study are available on reasonable request from the corresponding author on the condition of relevant approval from the originating institution.
